# A Method of Mounting a Porcelain-Faced Crown

**Published:** 1885-01

**Authors:** A. O. Hunt

**Affiliations:** Iowa City.


### AMERICAN JOURNAL

OF

### DENTAL SCIENCE.

VOL. XVIII. THIRD SERIES—JANUARY, 1885. No. 9.

ARTICLE I.

### A METHOD OF MOUNTING A PORCELAIN-FACED CROWN.

BY A. O. HUNT, D. D. S., IOWA CITY.

[Read before the Iowa State Dental Society, May 1884.]

One very important part of dental technics is that of crown setting.

There are many methods of doing this, some of which are very practical and easy of accomplishment when conditions are favorable.

When roots are crowded, the surrounding parts are subject to a class of diseases that are difficult to treat unless the pulp canal can be utilized, through which to pass the various therapeutic agents to be employed.

By the usual method of attaching a crown, it is necessary to remove it whenever treatment is to be resorted to Such removal means destruction to the crown ; as one well adjusted will in nearly all cases be so badly mutilated in the operation that it is much easier to make a new one than to restore an old.

Again, most of the methods are adapted to roots that are firm and after being cut off to the line of the gums, retain all their normal strength and form. It is often necessary to operate with roots whose buccal, lingual or lateral walls are disintegrated nearly or quite to the alveolar process.

The interior of the root may be coned out deeply, exposing frail and ragged edges, with the gum tissues overlying these edges.

The method I shall attempt to describe is one suited to these latter cases, *i. e.*, roots with frail and ragged edges, with the gum tissues overlying and filling the crown end. The following method will overcome most if not all of the difficulties in the way of success, and allow the setting of a crown that is durable, and useful. To get good access to the root, first pack it with soft gutta-percha pellets, pressing the material out over the edges and building on until the gum is displaced, so that when the gutta-percha is removed a large and free opening is had through the gum, and the edges of the root are fully exposed. This packing should remain two or three days. With a thin platinum ribbon (33 gauge) form a tube, place it over the end of the root and bind with fine wire, twisted until the platinum conforms to the shape of the root.

If the edges are too frail fill with amalgam in the following manner:

First roughen the inner side of the root or cut ledges if admissible with an engine burr. Select a broach that will enter the apical foramen. Leave this in the canal while inserting the amalgam. Restore the contour if lacking at the same time, and withdraw the broach carefully.

The channel thus formed will be a guide in drilling. Wait a day or two for the amalgam to harden; now the end of the root will present a firm face and shoulder, nearly as good as though the walls had never been destroyed.

With tube over root in place, prepare enough plaster (plaster two parts, sand or asbestos one part,) and take an impression of this and adjacent teeth, seeing that the plaster fills the tube down to the root.

When the impression is removed with the band, dry thoroughly. Pour into it Babditt metal, (Grade C,) or Watts or Reese metal.

Break away the plaster and remove the platinum tube, trim away in the metal that portion that corresponds to the gum tissue, leaving a correct model of the end of the root over which to place the permanent band of gold. Fit this tightly to the model, turning a flange over the labial edge, as a rest for the porcelain face. Now prepare the following: Hollow platinum wire (No. 60,) a twist drill a trifle smaller than the wire, cutting end cone shaped, so that it leaves the bottom of the drill-hole in that form.

Hard wax composed as follows: Gutta-percha one part, pure beeswax two parts, and sufficient resin to form a mass that will be hard at the temperature of the body. Platinum rolled foil (say No. 45 guage, to be used as backing for the crown.) Put the gold band in place over the root in the mouth, so that the flange is below the gum line.

Follow the pulp canal with the drill, securing as deep a socket as may be.

Select a suitable plain plate tooth (pins cross-wise preferred,) grind to fit, resting the cervical end on the flange of the band.

Lay a piece of the platinum foil on a soft wood surface and press the tooth pins through it. Burnish the foil down to the tooth and over the approximal, cutting the cervical edges so that at the latter point the foil will be between the tooth and the flange. Adjust the tooth so prepared to its place, driving the platinum tube or wire into the drill hole, allowing it to extend to the cutting edge of the tooth. With a hot instrument (flat burnisher,) melt hard wax about the tube, inside the band and on the backing of the tooth.

Arrange everything now as you would have it when finished. With plaster and sand, or asbestos, the same as before, take an impression. Should the tooth and band not draw out with it they can be removed and put in place in the impression.

Fill all with a mixture of plaster and sand. Remove the cup and trim down on the lingual side, exposing and removing all the wax. Bend the tooth pins up close to the tube, fill the tube with a piece of broom straw dipped in wet plaster.

Flow solder around these and down over the thin platinum backing until the whole is united to the band at the flange, and a good body of it on the backing. Fit a shell of gold the same grade as the band to the lingual side, resting on the band and fitting the backing closely at the approximal edges and around the tube, which should extend outside of the shell. Solder this in place and finish with files, etc., to a contour corresponding to the tooth being mounted.

Whatever of the tube projects, file off as surplus material.

Place the crown, when finished, in place, perfect the articulation. Remove and attach with cement (Caulk's Diamond preferred.) When mixed to the consistency of cream it gives ample time for the work, and sets hard and firm. Remove the pieces of broom corn and plaster from the tube; free access can thus be obtained through this channel, for treatment now or at any future time.

Fill the tube nearly to the end with cement, closing up with a gold filling.

The advantage of this method will be readily seen. It can be used on any tooth, in any part of the mouth.

In bicuspids with two roots, two tubes may be used; and the shell for the lingual side, formed over a piece of dry hickory or other hard wood or swaged over metal dies. In molars a tube may also be used for each root.

Another advantage is in having a hollow pin or wire, nearly the combined length of root and crown, firmly imbedded in cement, giving great strength with lightness. Another, the crown is easy of construction, requiring only those instruments usually found in an appointed dental office. Still another, it is durable; and the manipulation is postive and adapted to any shape of root.

In one case where this method was employed the root was but ⅜ of an inch in length (a superior second bicuspid) and quite loose, accompanied with ulceration. So frail that any method of band crowning would have been doubtful.

By this method and with good results in treatment after seventeen months of usage, the root is firm, the crown unimpaired, and all doing the service of a good tooth.—*Archives of Dentistry.*

---

ARTICLE II.

